# Neurophysiological Evidence of Sensory Prediction Errors Driving Speech Sensorimotor Adaptation

**DOI:** 10.1523/JNEUROSCI.2084-24.2025

**Published:** 2025-05-27

**Authors:** Kwang S. Kim, Leighton B. Hinkley, Kurtis Brent, Jessica L. Gaines, Alvincé L. Pongos, Saloni Gupta, Corby L. Dale, Srikantan S. Nagarajan, John F. Houde

**Affiliations:** ^1^Department of Speech, Language, and Hearing Sciences, Purdue University, West Lafayette, Indiana 47907; ^2^Department of Radiology and Biomedical Imaging, University of California San Francisco, San Francisco, California 94143; ^3^UC Berkeley - UCSF Graduate Program in Bioengineering, University of California San Francisco, San Francisco, California 94720; ^4^Department of Otolaryngology—Head and Neck Surgery, University of California San Francisco, San Francisco, California 94143

**Keywords:** motor control, motor learning, sensorimotor adaptation, sensory prediction error, speaking-induced suppression, speech production

## Abstract

The human sensorimotor system has a remarkable ability to learn movements from sensory experience. A prominent example is sensorimotor adaptation, learning that characterizes the sensorimotor system's response to persistent sensory errors by adjusting future movements to compensate for those errors. A component of sensorimotor adaptation is implicit (i.e., the learner is unaware of the learning) which has been suggested to result from sensory prediction errors—discrepancies between predicted sensory consequences of motor commands and actual sensory feedback. However, neurophysiological evidence that sensory prediction errors drive adaptation has never been directly demonstrated. Here, we examined prediction errors via magnetoencephalography imaging of the auditory cortex during sensorimotor adaptation of speech to altered auditory feedback, an entirely implicit adaptation task. Specifically, we measured how speaking-induced suppression (SIS)—a neural representation of auditory prediction errors—changed over the trials of the adaptation experiment. In both male and female speakers, reduction in SIS (reflecting larger prediction errors) during the early learning phase compared with the initial unaltered feedback phase positively correlated with behavioral adaptation extents, suggesting larger prediction errors were associated with more learning. In contrast, such a reduction in SIS was not found in a control experiment in which participants heard unaltered feedback and thus did not adapt. In addition, in some participants who reached a plateau in the late learning phase, SIS increased, demonstrating that prediction errors were minimal when there was no further adaptation. Together, these findings provide the first direct neurophysiological evidence for the hypothesis that prediction errors drive sensorimotor adaptation.

## Significance Statement

This work investigates mechanisms of sensorimotor adaptation, the phenomenon of motor learning due to exposure to altered sensory feedback. Models of motor control have hypothesized that sensorimotor adaptation is driven by sensory prediction errors—the discrepancy between predicted and actual sensory feedback. Here, we provide for the first time direct neurophysiological evidence that speech sensorimotor adaptation is indeed driven by sensory prediction errors using magnetoencephalography (MEG) imaging of auditory cortex during speaking.

## Introduction

The sensorimotor system shows a remarkable ability to quickly and efficiently learn movements based on sensory feedback. Soon after perceiving sensory errors that arise from movements, the system updates future movements to compensate for the errors, a phenomenon called sensorimotor adaptation. What drives such an elegant learning process? Previous studies suggested that adaptation can be driven by both task errors (explicit learning, i.e., driven by discrepancy between the action and the goal) and sensory prediction errors (implicit learning, i.e., driven by mismatches between the actual sensory consequences of a movement and those predicted from the motor commands driving that movement).

In the speech domain, however, multiple lines of evidence suggest that speech sensorimotor adaptation to altered auditory feedback may be driven mainly by sensory prediction errors given that the learning is implicit (i.e., participants are unaware of the learning and do not employ any strategy to learn, Kim and Max, 2021). In previous adaptation studies, participants showed no difference in the amount of learning in response to formant-perturbed auditory feedback when instructed to compensate, to ignore the feedback, or to avoid compensating (Munhall et al., 2009; Keough et al., 2013). Although behavioral studies have suggested that this unconscious minimizing of auditory prediction errors is the signal that drives speech sensorimotor adaptation, direct neurophysiological evidence of this process has not been demonstrated.

One neural representation of auditory prediction errors is speaking-induced suppression (SIS) of the auditory cortex. Studies have reported that the auditory responses to self-produced speech are smaller (i.e., suppressed) than the responses to playback of the same speech sound, consistent with the idea that auditory responses arise from auditory prediction errors, which are small in the self-produced case (i.e., auditory feedback is predictable) and large in the passively heard case (i.e., auditory feedback is unpredictable). Thus, SIS demonstrates that, during speaking, the auditory system predicts and anticipates the arrival of auditory feedback of speech onset, resulting in a suppressed feedback comparison response, as compared with auditory responses during passive listening to playback when speech onset cannot be predicted/anticipated. Consistent with the idea, SIS was reduced when participants spoke with pitch-perturbed auditory feedback (Behroozmand and Larson, 2011; Chang et al., 2013) or voice-manipulated auditory feedback (“alien voice”; Houde et al., 2002; Heinks-Maldonado et al., 2005, 2006). Importantly, this reduction in the suppression of auditory areas in response to perturbed auditory feedback are not unique to human speech, as they have also been observed in marmoset monkey vocal production (Eliades and Tsunada, 2018). In sum, in typical speaking conditions, a large SIS effect reflects minimal to none auditory prediction errors, and during altered feedback a reduction in SIS reflects an increase in prediction errors.

One previous study that examined SIS during adaptation to first formant frequency shifts via electroencephalography (EEG) reported that the average SIS amplitude across the learning phase (i.e., 80 trials with perturbed first formant) was not reduced compared with the preadaptation baseline average SIS (Sato and Shiller, 2018). However, most adaptation is known to occur in early trials (initial 20 to 40 perturbation trials, Kim and Max, 2021), raising the possibility that average SIS across all perturbation trials (as opposed to early trials) may mostly reflect neural activities after adaptation rather than activities that arise during adaptation. Here, we used magnetoencephalography (MEG) imaging during repeated speech adaptation sessions to test the hypotheses that (1) SIS reduces during early phases of speech sensorimotor adaptation and (2) the early SIS reduction may be distinct from SIS changes found in later phases of adaptation.

## Materials and Methods

### Participants

Participants who were native speakers of American English with no known communication, neurological, or psychological disorders were recruited for both MEG and MRI recording sessions. Thirty participants participated in the adaptation experiment, but eight participants were excluded from analyses for various reasons. One participant's source could not be reliably localized. Four participants could not finish the task due to fatigue. These participants’ data was excluded because they were not exposed to the same number of perturbed trials, making their adaptation data confounded by the lack of exposure to the perturbation. In addition, their data lack power for the evoked potential analyses. Two participants showed “following” nonadaptive behavior (i.e., change of 15 Hz or more in the direction of the perturbation in the late learning phase) and one participant had atypical (outlier) SIS response in the baseline, (SIS < –10 z).

Here, we report adaptation experiment results from the remaining 22 participants (mean age = 30.9, SD = 8.8 years old, 12 females). For the control experiment, 12 participants (mean age = 31.6, SD = 8.0 years old, 5 females) participated. It should be noted that five of these participants also participated in the adaptation experiment, each visit being 1–2 months apart. The order was pseudorandomized (i.e., three of the five participants did the adaptation experiment first). All participants in the study passed pure-tone hearing thresholds of ≤20 dB HL for the octave frequencies between 500 and 4,000 Hz, except one participant in the adaptation experiment whose threshold was at 30 dB in the right ear and 40 dB in the left ear at 4 kHz.

### Experimental design

#### Adaptation experiment

**Figure 1.**
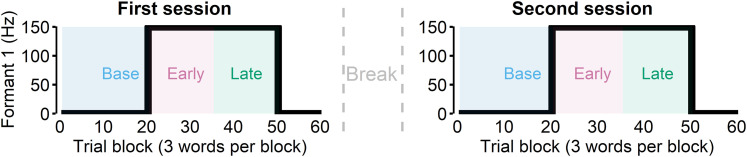
Participants were asked to read words during the first two sessions (“speak”). In these sessions, 150 Hz upshift perturbation was present from the trial block 21 to 50. We categorized the first 15 trial blocks of the perturbed trials (21–35) as the early learning phase and the second 15 trial blocks (36–50) as the late learning phase. After the first session, we asked participants to repeat another speaking session after a break lasting a few minutes.

Participants lay supine on the scanner bed of a whole-head, 275-channel biomagnetometer system (MEG; Omega 2000, CTF) for a total of four sessions (first and second speaking sessions, first and second listening sessions). The rationale for this repeated session was that most adaptation occurs quickly, often in the first 10–30 trials of the perturbation phase, but such a low number of trials does not provide enough power for the evoked potential analyses. Thus, to ensure an adequate number of trials for the early and late learning phases, an additional session was recorded. During the first two sessions, participants were asked to read “Ed,” “end,” or “ebb” (60 trial blocks for 3 different words = 180 total trials) that appeared on the screen. The order was randomized for each block of the three words. During these speaking sessions, participants heard their speech with the first formant frequency (Formant 1 or F1) shifted upward for some trials, which made their speech to sound more like “add,” “and,” and “abb,” respectively. Specifically, after the first 20 trial blocks (i.e., baseline) which had no perturbation, the 150 Hz upshift perturbation was applied through Feedback Utility for Speech Processing (FUSP; Kothare et al., 2020) from trial block 21 to 50. The total feedback latency (i.e., hardware + software; Kim et al., 2020) was measured to be ∼19 ms. We categorized the first 15 trial blocks of the perturbed trials (21–35) as the early learning phase and the second 15 trial blocks (36–50) as the late learning phase (Fig. 1).

Two speaking sessions were followed by two listening sessions during which participants listened to the same auditory feedback recorded from the speaking sessions. It should be noted that during the listening sessions, participants were provided with the same auditory input they previously heard (i.e., recordings of auditory feedback) which included perturbed auditory feedback during the perturbation phase. During the listening sessions, participants also saw the same visual stimuli that they saw (i.e., same words) in the speaking sessions. Between each session, participants were given a few minute-long break that included conversations with the experimenter, which allowed additional exposure to their unaltered auditory feedback. Given that the adaptation task (i.e., speaking session) was repeated, we also checked whether there was any savings effect and found that there was no consistent effect of repeating adaptation in both early learning, *t*_(21)_ = –0.830, *p*_adj_ = 0.416, and late learning, *t*_(21)_ = 1.062, *p*_adj_ = 0.300.

#### Control experiment

We also designed a control experiment in which we applied 0 Hz perturbation (instead of 150 Hz perturbation) during early and late “learning” phases. All other details of the task remained identical to the adaptation experiment.

#### MRI

On a separate day, participants also underwent an MRI scan, where a high-resolution T1-weighted anatomical MRI (3.0T Siemens Trio; MPRAGE; 160 1 mm slices; FOV, 256 mm; TR, 2,300 ms; TE, 2.98 ms) was acquired in each participant for source reconstruction.

### MEG acquisition

Participants were placed in a 275-channel, whole-head biomagnetometer system (Omega 2000, CTF; sampling rate, 1,200 Hz; acquisition filtering, 0.001–300 Hz) for a total of four sessions (two speaking and two listening sessions). Participants heard auditory feedback (or recorded auditory feedback during listening condition) through ER-3A ear-insert earphones (Etymotic Research) and a passive fiber optic microphone (Phone-Or) was placed about an inch in front of their mouths to record speech responses. All stimulus and response events were integrated in real time with MEG time-series via analog-to-digital input to the imaging acquisition software.

Each participant lay supine with their head supported inside the helmet along the center of the sensor array. Three localizer coils affixed to the nasion, left peri-auricular, and right peri-auricular points determined head positioning relative to the sensor array both before and after each block of trials. We ensured that participants’ head movements were smaller than 5 mm in every session. Coregistration of MEG data to each individual's MRI image was performed using the CTF software suite (MISL; ctfmeg.com; version 5.2.1) by aligning the localizer coil locations to the corresponding fiducial points on the individual's MRI. MRI images were exported to Analyze format and spatially normalized to the standard T1 Montreal Neurological Institute (MNI) template via Statistical Parametric Mapping (SPM8, Wellcome Trust Centre for Neuroimaging).

### Data extraction and analyses

#### Acoustic analysis: estimating first formant frequency (F1)

The first formant frequency (F1) from each speech production was extracted through a custom MATLAB software, Wave Viewer (Raharjo et al., 2021). We then extracted F1 from the vowel midpoint (40–60% into the vowel) and averaged it for each utterance. In case of missing trials, we replaced the data point by using an interpolation method using four nearest neighboring trials as described in Kitchen et al. (2022). We replaced ∼2.96 and 2.88% of the data for the adaptation and control experiments, respectively. We normalized the data by subtracting the baseline F1 from the data (i.e., baseline = 6th to 20th trial blocks). The amount of learning in each phase was assessed by averaging the last five trial blocks (31st–35th blocks for early learning and 46th–50th blocks for late learning).

#### Speaking-induced suppression

We first corrected distant magnetic field disturbances by calculating a synthetic third-order gradiometer, detrended using a DC offset across whole trials, and then filtered (fourth-order Butterworth, bandpass 4–40 Hz) sensor data. In the sensor data of two participants (one of them participated in both of the experiments, so three datasets), considerable (>10pT) sensor noise caused by dental artifact verified through visual inspection was denoised using a dual signal subspace projection (DSSP; Cai et al., 2019a,b). After preprocessing sensor data, separate datasets were created with trials during baseline, early learning, and late learning phases for speak and listen conditions. In these datasets, trials exceeding a 2 pT threshold at any timepoint were rejected. In two participants’ data, three channels were removed prior to threshold-based artifact rejection. The data was then averaged across all remaining channels. For the adaptation experiment, 5.47% of the speak session trials and 4.70% of the listen session trials were removed. For the control experiment, 5.68 and 6.14% of the trials were removed for speak and listen sessions, respectively.

For each participant, a single-sphere head model was derived from the individual's coregistered T1 structural MRI using the CTF software suite (MISL; ctfmeg.com; version 5.2.1). Using the Champagne algorithm (Owen et al., 2012) and a lead field of 8 mm resolution on the baseline listen data, we generated whole-brain evoked activity between 75 and 150 ms (after the auditory feedback onset) and determined the MNI coordinate with the most pronounced M100 response in the left and right auditory areas (i.e., the highest amplitude) for each participant. The median MNI coordinate across adaptation and control experiments were [*x* = –56, *y* = –24, *z* = 8] and [*x* = 48, *y* = –24, *z* = 8] for the left and right auditory areas, respectively. We then used a Bayesian adaptive beamformer (Cai et al., 2023) to extract time-series source activity focused on the obtained MNI coordinate across all phases (i.e., baseline, early, and late). From the final time-series *z*-scored data, we measured M100 peak by finding the maximum value between 75 and 150 ms after the auditory signal. We then computed the M100 amplitude difference between the listen and speak sessions to determine SIS:

$$\mathrm{SIS}\;=\;M100_{\mathrm{listen}}-M100_{\mathrm{speak}}.$$

**Figure 2.**
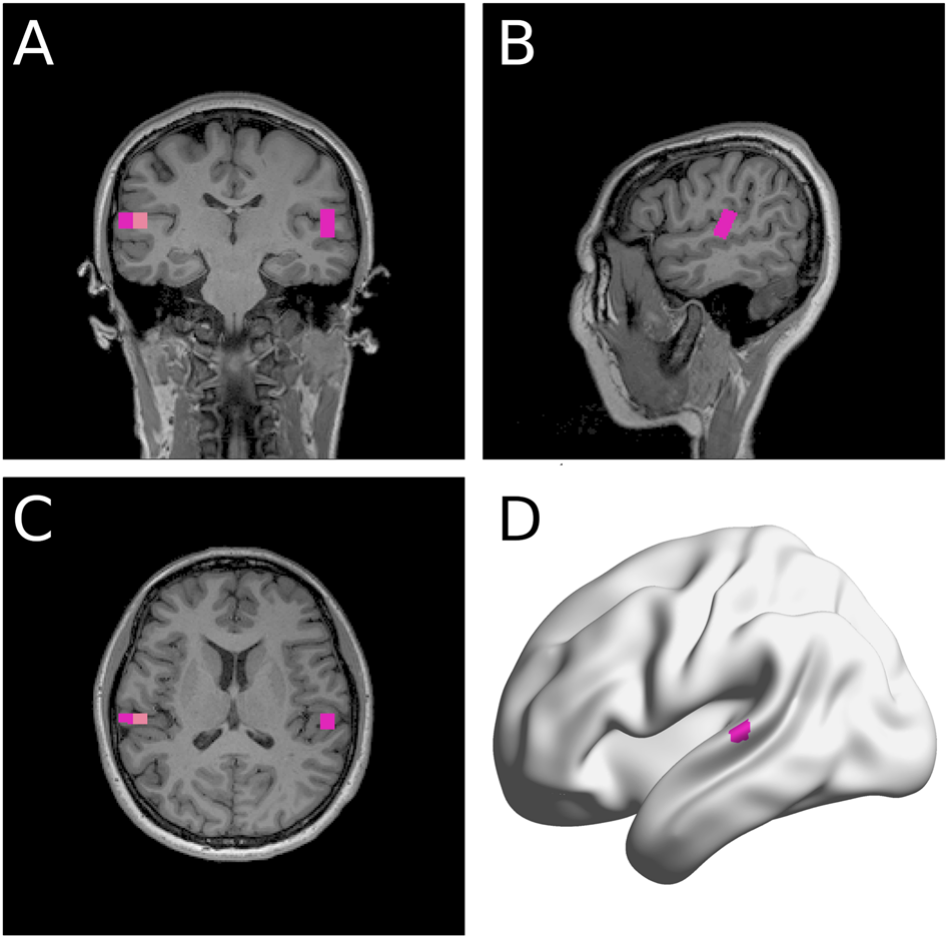
A representative participant's source localization. NUTMEG (Hinkley et al., 2020) identified a few MNI coordinates that showed clear M100 response shown in the coronal (***A***), sagittal (***B***), and transverse (***C***) planes. The MNI coordinate of the voxel with the most power in the auditory areas in each hemisphere was selected for analyses. ***D***, The same participant's left auditory area coordinate selected shown in a surface-based rendering (BrainNet Viewer; Xia et al., 2013).

We averaged the acoustic and MEG data across the repeated sessions. As shown in Figure 2, source localization of trial-averaged data for each condition (speak, listen) and phase (baseline, early learning, and late learning) was conducted to determine peak activity (M100) location within the auditory cortex. We then computed the M100 amplitude differences between the listen and speak sessions to determine SIS for each condition and phase. It should be noted that because the acoustics of the auditory feedback changes during adaptation (due to the auditory perturbation), we compared M100 across speak versus listen conditions for each corresponding phase (i.e., baseline listen M100–baseline speak M100).

### Statistical analyses

A linear mixed effects model was constructed for SIS with the different adaptation phases as fixed effects and participants as a random effect using the *lme4* package in R (Bates et al., 2015). Tukey’s test was used for post hoc pairwise comparisons from the *emmeans* package in R (Lenth, 2022), which applies *p* value corrections to each set of comparisons of the means (i.e., *p*_adj_). In addition, a Pearson's correlation tested to examine relationships between the amount of adaptation and the SIS amplitudes. For correlation tests, we also performed the approximate correlation power calculation via Fisher's z-transformation using the *pwr* package in R (Champely et al., 2020) based on the correlation coefficient (*r*), significance level (*α*), and sample size (*n*).

## Results

**Figure 3.**
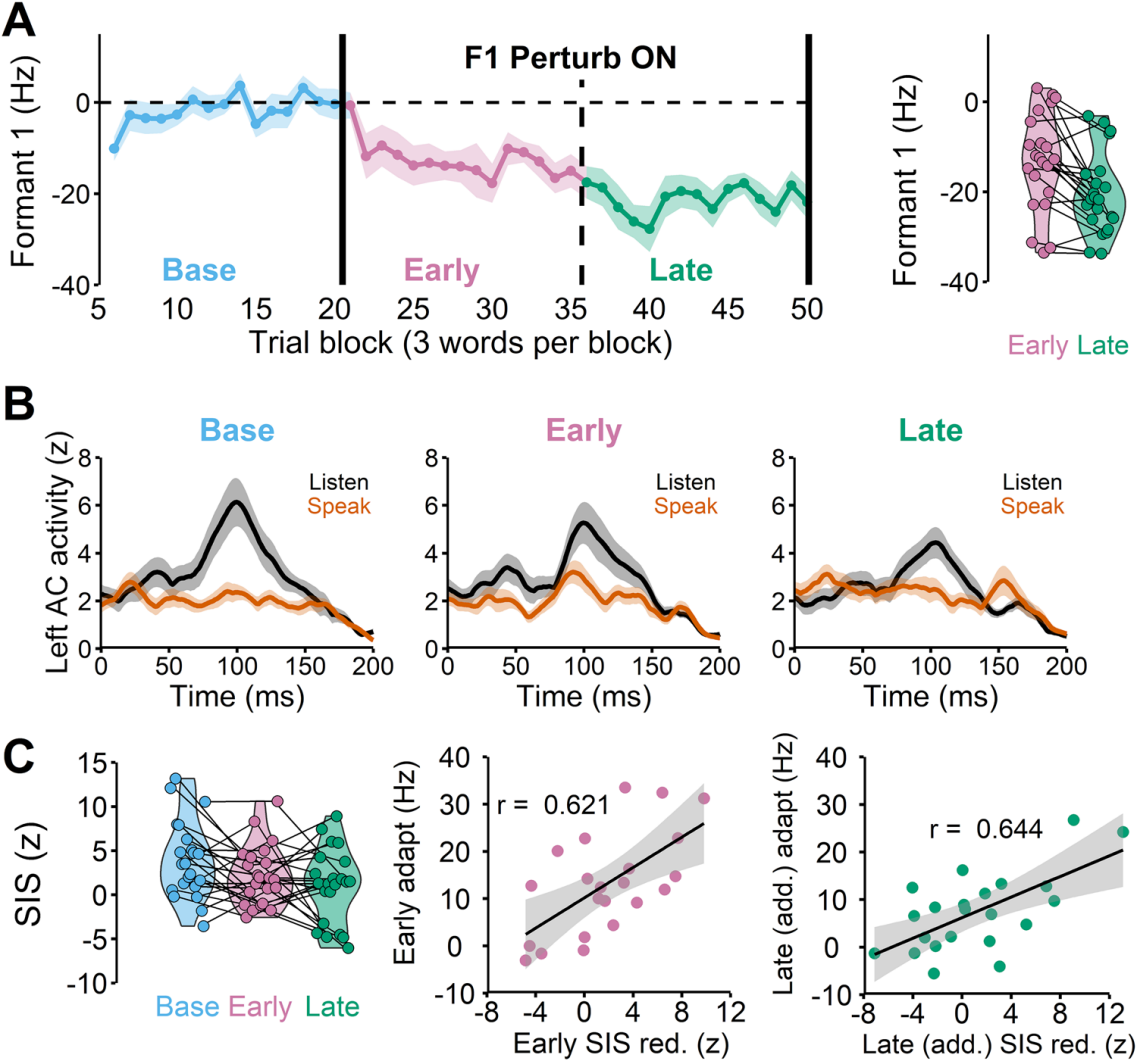
***A***, The group average speech auditory-motor adaptation in which participants lowered their first formant frequency (F1) in response to the 150 Hz upshift F1 perturbation (left). Each participant's early and late adaptation. Some participants continued to adapt after the early phase, but others plateaued (right). ***B***, The left auditory cortex responses (M100) in listen and speak conditions demonstrate that the amount of speaking-induced suppression [i.e., listen (black)—speak (orange)] is reduced during early learning (Early) compared with the baseline (Base). ***C***, SIS was significantly reduced in the early and late learning phases compared with the baseline (left, *r*(20) = 0.621, *p* = 0.002). The amount of SIS reduction in the early learning phase was significantly correlated with the amount of early adaptation (middle). The amount of additional SIS reduction in the late learning phase also significantly correlated with the additional amount of adaptation in the phase (right, *r*(20) = 0.644, *p* = 0.001).

On average, participants adapted by lowering their F1 by 20 Hz in the speaking sessions (Fig. 3*A*). Most participants also showed a clear suppression of left auditory activity in the speaking condition (compared with the listening condition) during the baseline phase (Fig. 3*B*, left), unlike SIS in the right hemisphere. Hence, SIS refers to suppression of left auditory activity hereafter unless specified otherwise.

We found that the SIS response changed across the baseline, early, and late learning phases (Fig. 3*B*, middle and right), *F*_(2,44)_ = 4.788, *p* = 0.013. The post hoc pairwise-comparison test indicated that SIS response in the early learning phase did not differ from the baseline, *t*_(46.1)_ = 1.829, *p*_adj_ = 0.171 due to the wide range of inter-individual variability (Fig. 3*C*, left). Interestingly, we found that participants who adapted more (in the early learning phase) also had a greater amount of early SIS reduction, *r*(20) = 0.621, *p* = 0.002 (Fig. 3*C*, middle). The approximate power based on the coefficient (*r* = 0.621), significance level (*α* = 0.05), and number of observations (*n* = 22) was 0.897.

In the late learning phase, the SIS amplitude was significantly reduced compared with the baseline (Fig. 3*C*, left), *t*_(46.1)_ = 4.339, *p*_adj_ = 0.002. We found that the SIS reduction from the baseline was not significantly correlated, though trending, with the final amount of adaptation in the late learning phase, *r*(20) = –0.416, *p* = 0.054. This result was somewhat consistent with our hypothesis that most learning typically occurs in the early phase, and thus the late phase SIS reduction from baseline would not be able to capture most of the adaptation extent. Rather, late SIS reduction that accounts for early SIS changes (i.e., additional late SIS reduction from early SIS) is likely a predictor for late (additional) learning behaviors. Indeed, we found that additional SIS reduction in the late learning phase (i.e., late SIS relative to the early SIS) was significantly correlated with additional late adaptation, i.e., late adaptation relative to early adaptation *r*(20) = 0.644, *p* = 0.001. For this correlation result (*r* = 0.644, *α* = 0.05, *n* = 22), the approximate power was 0.924.

Taken together, the relationship between additional SIS reduction and adaptation in the late learning phase also followed the same trend found in the early learning phase. That is, individuals who showed more reduction in SIS also tended to show more learning, suggesting that larger adaptation was associated with larger prediction errors. In contrast, less learning or no learning behavior (e.g., reaching a plateau) was associated with smaller prediction errors (i.e., increases in SIS).

### SIS remained unchanged when there was no learning

**Figure 4.**
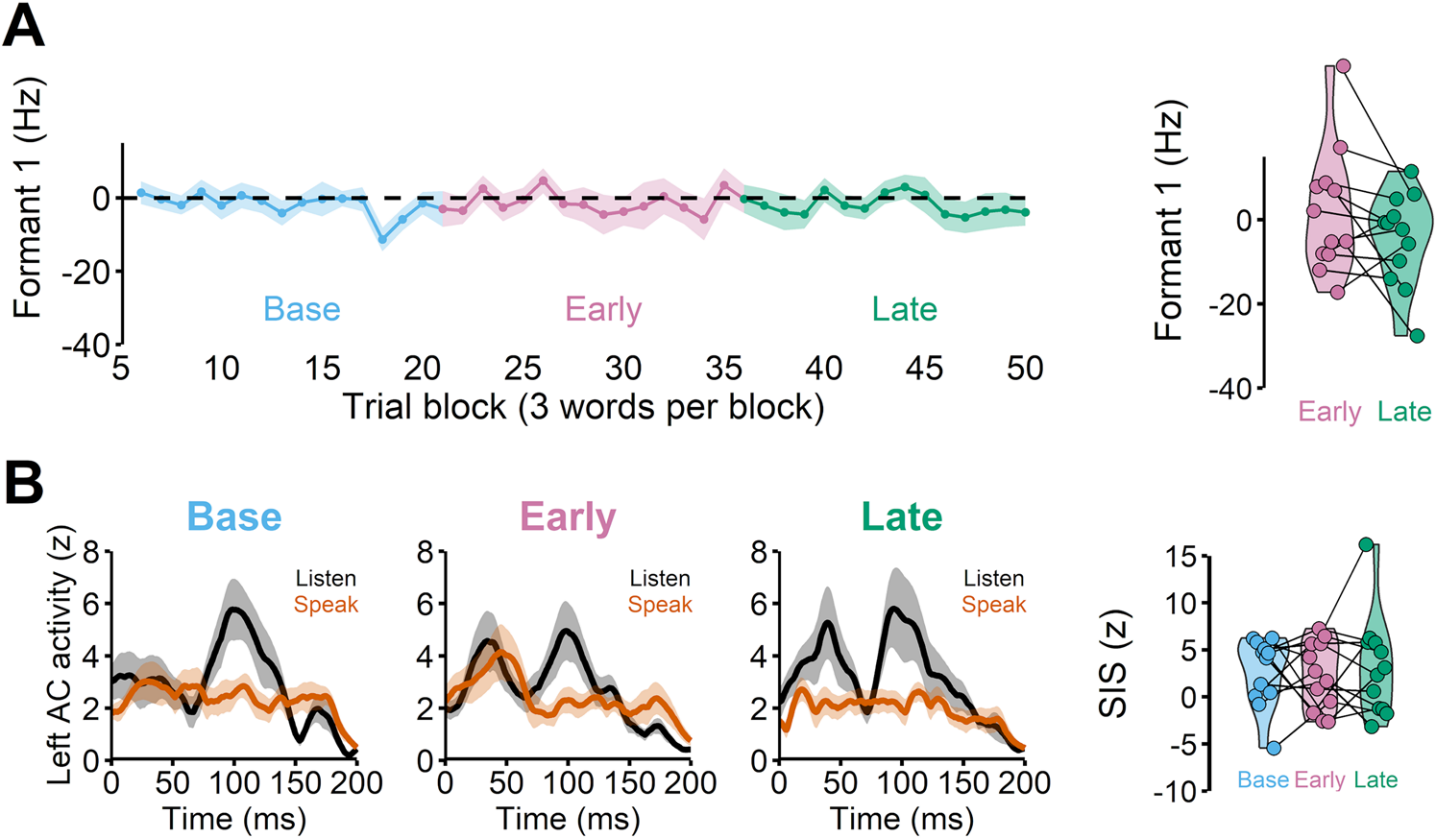
A control experiment in which no auditory perturbation was applied. ***A***, Participants did not show any changes in Formant 1, exhibiting no learning (also see the top right panel for individual F1 data). ***B***, There was also no SIS change across the different phases.

To ensure that SIS reduction was related to learning behaviors, we designed a control experiment in which there was no auditory perturbation (and thus no learning was expected). Here, participants also completed two speaking and two listening sessions. Other than the absence of the perturbation, the experimental setup and the analyses methods were identical to the main experiment. We found that participants did not adapt (Fig. 4*A*) and SIS reduction also did not occur (i.e., SIS amplitudes did not change across the phases), *F*_(2,24)_ = 0.211, *p* = 0.812. Therefore, SIS remained unchanged when there was no learning.

### Right hemisphere SIS remained unchanged

**Figure 5.**
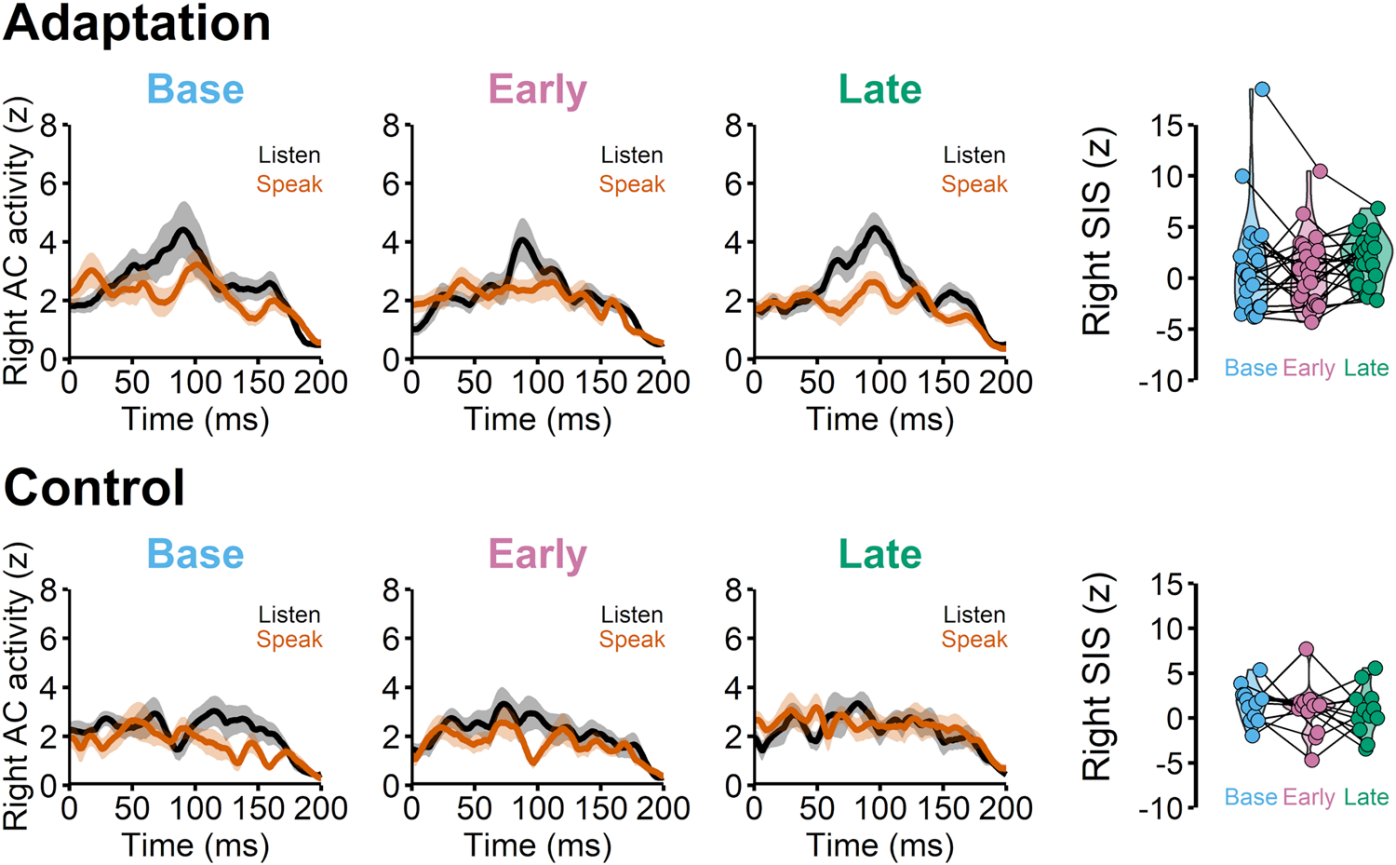
Right hemisphere SIS data for the adaptation experiment and control experiment. Top row, We did not find a significant SIS effect during the baseline in the right hemisphere in the adaptation group, *t*_(21)_ = 1.148, *p* = 0.264. There was also no significant SIS reduction across the different phases, *F*_(2, 44)_ = 0.934, *p* = 0.401. Bottom row, Although the baseline SIS was significant in the control experiment, *t*_(11)_ = 2.780, *p* = 0.018, we also did not observe any significant right hemisphere SIS changes across the phases in the control experiment, *F*_(2,24)_ = 0.469, *p* = 0.631.

Compared with the left auditory cortex, the right hemisphere SIS activities were less pronounced. Multiple individuals did not show a clear SIS response in the right hemisphere even during the baseline in the adaptation group, *t*_(21)_ = 1.148, *p* = 0.264 (Fig. 5, left). In the control group, however, SIS was significant, *t*_(11)_ = 2.780, *p* = 0.018 (Fig. 5, right). In the adaptation group, we did not observe a significant SIS reduction in adaptation phases in the right auditory cortex, *F*_(2, 44)_ = 0.934, *p* = 0.401, in line with a previous study that found prediction-related SIS effect only in the left hemisphere (Niziolek et al., 2013). We also did not find any significant SIS reduction in the control group's right hemisphere activities, *F*_(2, 24)_ = 0.469, *p* = 0.631.

## Discussion

We used magnetoencephalography (MEG) imaging to examine auditory prediction errors during speech auditory-motor adaptation. Specifically, we measured speaking-induced suppression (SIS)—suppression of auditory responses to self-produced speech compared with the responses to passively heard speech—which is thought to represent auditory prediction errors. To fully capture SIS changes in the early learning phase during which most of adaptation typically occurs, we analyzed the early learning and late learning phases separately.

### Neurophysiological evidence that auditory prediction errors drive implicit adaptation

SIS was significantly reduced in the early learning phase during which adaptation occurred. In contrast, in a control experiment in which there was no perturbation (and thus no adaptation), such an SIS reduction was not found. In addition, the amount of SIS reduction was positively correlated with the amount of adaptation, delineating a direct link between prediction errors and adaptation. Furthermore, in the late learning phase, SIS increase, instead of SIS reduction, was associated with adaptation reaching an asymptote (i.e., absence of further learning). Hence, it is unlikely that SIS change arises simply because of adaptive behavior (i.e., lower F1 productions). Rather, SIS reduction likely reflects prediction error signals that lead to adaptation. In sum, our results suggest that auditory prediction errors drive speech auditory-motor adaptation.

Our findings are consistent with previous reports of speech adaptation being entirely implicit (Lametti et al., 2020; Kim and Max, 2021), which is thought to be driven by prediction errors (Mazzoni and Krakauer, 2006; Haith and Krakauer, 2013). In addition, speech adaptation also seems to be sensitive to auditory feedback delays (i.e., 100 ms delay can eliminate adaptation), which highlights the importance of prediction errors that require temporally precise comparison of prediction and the actual feedback (Max and Maffett, 2015; Shiller et al., 2020). More recently, a computational model, Feedback-Aware Control of Tasks in Speech (FACTS; Parrell et al., 2019) also generated simulations of adaptation driven by auditory prediction errors (Kim et al., 2023). Recently, Tang et al. (2025) showed that SIS was changed after exposure to auditory perturbation that manipulated participants’ perceived variability. Given that this type of learning during which participants change production variability (Tang et al., 2022; Wang and Max, 2022) likely involves prediction errors as in the current study, the results of Tang et al. (2025) are consistent with our findings.

To date, only one other study examined SIS during speech auditory-motor adaptation and found that SIS in P2 response correlated with adaptation (Sato and Shiller, 2018). Although that study did not find N1 SIS changes across adaptation, somewhat inconsistent with our finding, there are notable differences between studies in both the methods used to acquire data and subsequent analyses that may have contributed to the discrepancies. In the previous study, EEG data across the whole learning phase was averaged together, which likely included SIS recovery response in the late phase as found in the current study. Hence, it is possible that SIS reduction was present in the early learning phase, but such an effect may have been weakened by inclusion of late learning data. Additionally, in contrast to the sensor-level analysis of the previous study that includes contributions from both hemispheres, our study applied source localization via the Champagne algorithm (Owen et al., 2012) which allows robust estimation of the activity in the auditory cortex and separate analyses for each hemisphere.

Broadly, our findings provide the first neurophysiological evidence that sensory prediction errors drive implicit adaptation in humans. A similar suppression effect has been previously documented in the cerebellum of rhesus monkey during head movement adaptation (Brooks et al., 2015). In the study, cerebellar neuron activities, which are typically suppressed during voluntary movements compared with passive movements much like SIS, did not differ between the two conditions (voluntary vs passive) during adaptation. Remarkably, this reduced suppression also recovered (i.e., suppression increased) toward later learning trials, directly in line with our result. Here, we expanded the previous finding by demonstrating that the extent of such suppression reduction (or recovery) was closely associated with implicit adaptation across individuals.

### Adaptation plateaus when prediction errors are minimal

Another interesting finding of the current study concerns a potential mechanism that causes adaptation to halt. It has been reported that speech adaptation plateaus with a largely incomplete extent (see Kitchen et al., 2022 for further discussion). In the current study, late learning phase SIS increased (i.e., minimal prediction errors) in multiple participants who also showed plateaued adaptation in the phase (i.e., no additional learning), a finding consistent with the idea that sensory forward model updates (i.e., prediction updates) may have occurred throughout adaptation, minimizing prediction errors.

This idea that adaptation is halted by minimal prediction errors was also put forth by our computational model, FACTS (Kim et al., 2023). In these simulations, the adaptive motor output produced lower F1 in response to F1 upshift perturbation, resulting in perturbed sensory feedback to become more like the baseline sensory feedback. Interestingly, the simulations showed that sensory prediction was also updated to predict perturbed auditory feedback (i.e., higher prediction in F1). Thus, prediction errors, the difference between lower perturbed feedback in F1 and higher prediction in F1, became minimized throughout adaptation (i.e., because of both the motor output changes and sensory prediction updates), eventually becoming a small amount that could no longer induce adaptation.

Empirical evidence for the idea that minimal prediction errors may result in halting adaptation can also be found in head movement adaptation of rhesus monkeys (Brooks et al., 2015). In the study, cerebellar neuron activities to the voluntary head movement became more suppressed (compared with passive movement) as adaptation plateaued. Critically, the authors argued that the neural response becoming more suppressed throughout learning demonstrates that sensory prediction was being rapidly updated to predict unexpected sensory perturbation. Thus, our findings corroborate the notion that incomplete adaptation may result from not only the motor output changes but also sensory prediction updates, which together minimize prediction errors.

### What does SIS reflect?

SIS is typically viewed as a measure that reflects prediction errors given that SIS is reduced by auditory perturbations yield mismatch between prediction and feedback (Heinks-Maldonado et al., 2005). In contrast to this view, a previous study from our laboratory argued that the SIS response may instead reflect target errors, discrepancies between an intended auditory target with auditory feedback (Niziolek et al., 2013). However, our finding that SIS increased in nine participants during late learning cannot be easily explained by this account. Due to the SIS recovery, their late learning phase SIS response did not differ from their baseline SIS, which would be interpreted as minimal or no target errors according to the target error explanation for SIS. Nonetheless, these participants compensated for only 13.6% of the perturbation on average, presumably leaving a considerable discrepancy between any fixed auditory target and auditory feedback.

Alternatively, if SIS indeed reflects prediction errors rather than target errors, this view offers a different interpretation of Niziolek et al. (2013). According to the view, reduced SIS in productions with greater deviations from the median production may have resulted from large signal-dependent noise that stemmed from both the lower neural and muscular motor systems (Harris and Wolpert, 1998; Jones et al., 2002; Houde et al., 2014). Because such noise cannot be predicted by cortical areas, observed auditory feedback would not match auditory prediction, leading to large prediction errors. Hence, it is plausible that the reduced SIS found in those productions reflects larger prediction errors.

It should be noted that sensory attenuation/modulation that occurs prior to movement onset may be associated with predictions about future actions (Voss et al., 2008) and/or motor planning (Li et al., 2024). SIS, on the other hand, results from auditory response to ongoing speech, thus likely reflecting more sensory-level prediction error information.

### Neural correlates of auditory prediction errors

In the current study, we estimated auditory prediction errors from activities in the auditory cortex. Given that auditory areas receive corollary discharge from speech motor areas during speech production (Khalilian-Gourtani et al., 2024), it is possible that auditory prediction errors may be computed in the auditory cortex. However, a large body of evidence suggests that the cerebellum may be a neural substrate for forward models that generate sensory predictions (Wolpert et al., 1998; Blakemore et al., 1999, 2001; Kawato et al., 2003; Pasalar et al., 2006; Shadmehr and Krakauer, 2008; Imamizu and Kawato, 2012; Therrien and Bastian, 2019; Skipper and Lametti, 2021). Studies have also documented evidence that the cerebellum may also compute sensory prediction errors (Blakemore et al., 2001; Brooks et al., 2015; Cullen and Brooks, 2015).

Alternatively, it has also been hypothesized that the cerebellum may work in concert with cortical areas to generate prediction errors (Blakemore and Sirigu, 2003; Haar and Donchin, 2020). The cerebellum's projection to the posterior parietal cortex (Clower et al., 2001) has been implicated for generating sensory prediction (Desmurget and Grafton, 2000 ; Della-Maggiore et al., 2004). Indeed, some studies have reported auditory fibers projecting from the superior temporal gyrus and higher-order auditory regions to the cerebellum in primates (Brodal, 1979).
